# Association between hypertensive disorders of pregnancy and risk of autism in offspring: a systematic review and meta-analysis of observational studies

**DOI:** 10.18632/oncotarget.23030

**Published:** 2017-12-07

**Authors:** Ruo-Ting Xu, Qing-Xian Chang, Qi-Qiong Wang, Jian Zhang, Lai-Xin Xia, Nanbert Zhong, Yan-Hong Yu, Mei Zhong, Qi-Tao Huang

**Affiliations:** ^1^ Department of Neurology, Nanfang Hospital, Southern Medical University, Guangzhou, China; ^2^ First School of Clinical Medicine, Southern Medical University, Guangzhou, China; ^3^ Department of Obstetrics and Gynecology, Nanfang Hospital, Southern Medical University, Guangzhou, China; ^4^ Department of Neonatology, Nanfang Hospital, Southern Medical University, Guangzhou, China; ^5^ Department of Medical Genetics, School of Basic Medical Sciences, Southern Medical University, Guangzhou, China; ^6^ Department of Developmental Biology, School of Basic Medical Sciences, Southern Medical University, Guangzhou, China; ^7^ March of Dimes Global Network for Maternal and Infant Health, White Plains, NY, USA; ^8^ Department of Human Genetics, New York State Institute for Basic Research in Developmental Disabilities, Staten Island, NY, USA

**Keywords:** autism, childhood, pre-eclampsia, hypertensive disorders of pregnancy

## Abstract

**Background:**

Autism spectrum disorder (ASD) is a common severe pervasive neurodevelopmental disorder of undetermined etiology. Environmental exposures, especially pregnancy complications, have been increasingly recognized as a potential risk factor for ASD. Our aim was to (1) systematically evaluate the association between hypertensive disorders of pregnancy (HDP) and the risk of ASD in offspring, (2) specifically draw a subgroup analysis of disease severity in patients with HDP to achieve more sufficient evidence on this issue.

**Results:**

A total of 21 studies were identified with more than 6.5 million participants, including 31,027 ASD probands. A comparative meta-analysis established that offspring born premature to HDP were significantly associated with ASD than matched controls (OR = 1.42, 95% CI: 1.34–1.50). Subgroup analysis of clinical classification include: (1) gestational hypertension, (2) pre-eclampsia, (3) chronic hypertension complicating pregnancy (CHP). The offspring of mothers with pre-eclampsia and CHP have slightly higher risk (OR = 1.43; OR = 1.48, respectively) of ASD than those of mothers with gestational hypertension (OR = 1.37). In consistence with most previous researches, higher ASD prevalence was observed in male than female (OR = 1.38), indicating a potential role for gender in the pathophysiology of ASD.

**Materials and Methods:**

We conducted a systematic literature search on PubMed, EMBASE, Web of Science, PsycINFO database and China National Knowledge Infrastructure up to Jun. 2017. Statistical analysis was performed using Stata 10.0.

**Conclusions:**

This meta-analysis implies a possible link between HDP and the risk of ASD in offspring. However, further investigation should be conducted to confirm this conclusion, and intensive prenatal surveillance and early prediction for ASD is needed.

## INTRODUCTION

Autism spectrum disorders (ASD) is a neurodevelopmental syndrome characterized by various degrees of social impairment, deficits in language and communication, and repetitive patterns of behavior [1, 2], but the underlying mechanisms remain to be elucidated. The reported prevalence of ASD has increased dramatically over time to approximately 100/10,000 (1%) today [3, 4], affecting the lives of 700,000 people and costing £32 billion each year in the UK [5]. The influence of prenatal exposures on development of ASD is gradually recognized, since substantial advances have been achieved in understanding the neurodevelopmental consequences of intrauterine challenges [6].

Hypertensive disorders of pregnancy (HDP) is a complex multisystem disorder, encompassing (1) gestational hypertension, (2) chronic hypertension complicating pregnancy (CHP), (3) pre-eclampsia and eclampsia [7], which can lead to severe maternal and fetal morbidity and even mortality [8]. Numerous studies have shown that fetal exposed to HDP had increased susceptibility to multiple neurodevelopmental disorders such as cognitive impairment [9] , depression [10], schizophrenia [11], and even elevated lifetime risks for stroke [12]. Ratsep et al. [13] reported that offspring of pre-eclamptic pregnancies exhibited altered brain structural and vascular anatomy as enlarged brain regional volumes of the cerebellum was detected by magnetic resonance imaging, which shared similarities with those seen in ASD. It is demonstrated that pregnancies affected by pre-eclampsia are correlated with an exaggerated immune responses, creating a chronic and uncontrolled state of inflammation [14, 15]. Epidemiological studies suggest that maternal infections (such as parasitic, bacterial and viral infection) [16] and autoimmune disorders (such as rheumatoid arthritis, asthma, systemic lupus erythematosis) [17], all of which lead to elevated immune responses, have been confirmed as independent risk factors for ASD [18].

Although a number of clinical studies [19–38] have been conducted to explore the undetermined association between HDP and ASD, the results of investigations were rather heterogeneous without collectively comparison. For instance, Walker et al. [26] observed that the children with ASD were twice as likely to have been exposed in utero to pre-eclampsia with controls, while Langridge et al. [36] failed to demonstrate statistically significant association between them. To determine whether all recent published epidemiologic studies, in combination, support an association between maternal HDP and the risk of ASD in offspring, we conducted a systematic review and meta-analysis of this issue. The aims of this study were to estimate the summary odds ratio for this association and have a comprehensive subgroup analysis to achieve more sufficient evidence of the association between disease severity and the risk of ASD.

## MATERIALS AND METHODS

This meta-analysis was conducted following the guidance provided in the Cochrane Handbook and was reported according to the Meta-analysis of Observational Studies in Epidemiology (MOOSE) guidelines [39].

### Search strategy

**Figure 1:**
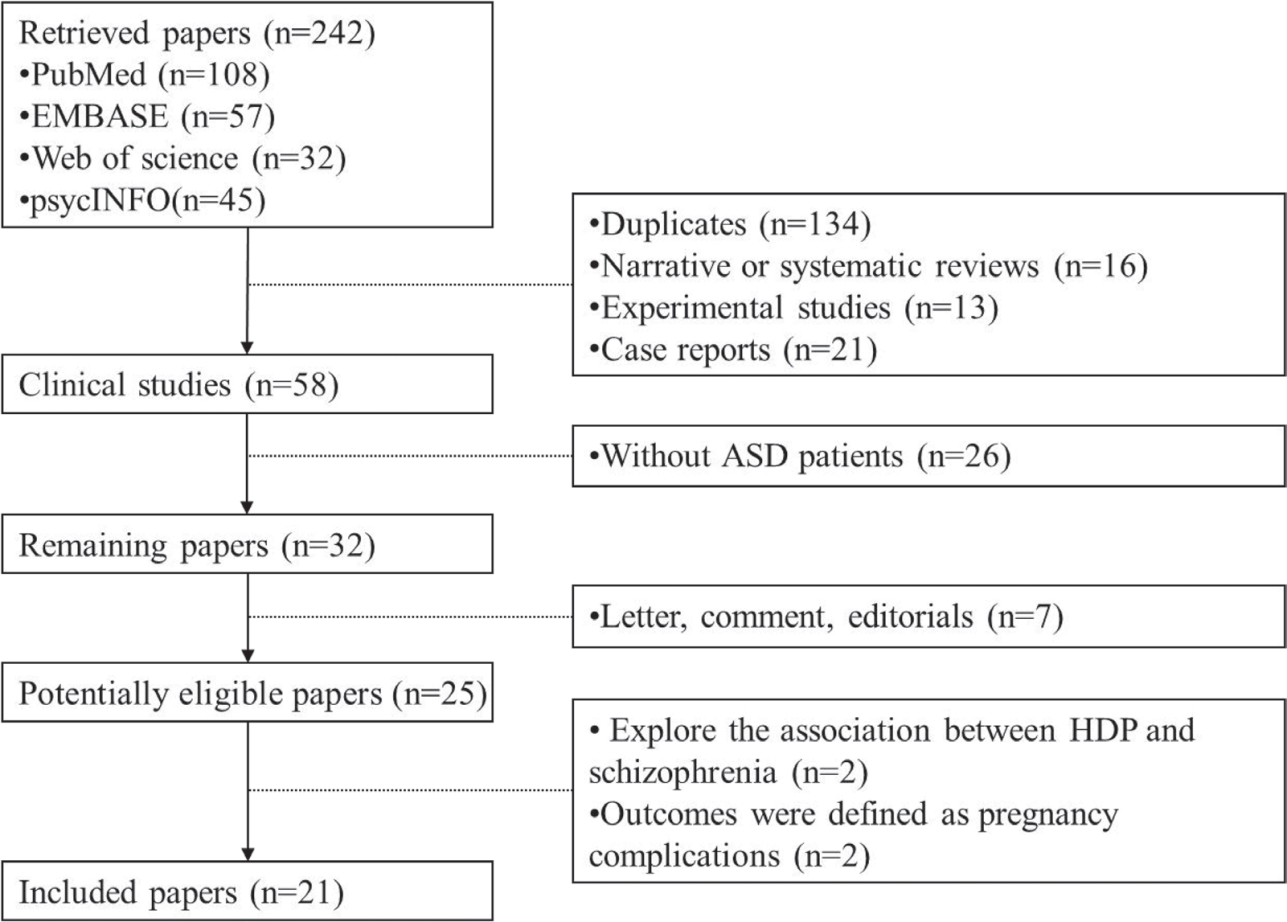
Flow diagram of the studies selection process

We performed a systematic electronic search in all available literature until Jun. 2017 on PubMed, EMBASE, Web of Science, PsycINFO database and China National Knowledge Infrastructure. We searched with the terms: “preeclampsia”, “HDP”, “hypertensive disorders of pregnancy”, “hypertensive disorders in pregnancy”, “hypertensive disorders complicating pregnancy”, “pregnancy hypertension”, “PIH”, “pregnancy induced hypertension”, “gestational hypertension”, “gravid hypertension”, “gravidic hypertension”, “ASD”, “autism spectrum disorder”, “autism”, “autistic disorder”, “pervasive development disorders”, “children”, “childhood”, “infantile”. Searches were limited to human articles published in the English or Chinese. Two investigators inspected the titles and abstracts of citations and obtained the full texts. The search strategies are summarized in Figure 1. We also searched for additional relevant studies by browsing the bibliographies of the included trials and related reviews.

### Inclusion and exclusion criteria

Studies were eligible if they: (i) were case–control studies, prospective studies, or RCTs; (ii) evaluated the association between HDP and ASD in offspring; (iii) presented relative risk (RR) or odds ratio (OR) estimates with 95% confidence intervals (CI) or necessary data for determination. Studies were excluded if they were (a) review articles, case reports, experimental studies, conference abstracts, animal studies or letters; (b) unpublished data; (c) lacked essential data for the pooled calculation. Two reviewers independently evaluated titles and abstracts of the identified articles and subsequently excluded those that were irrelevant.

### Data extraction

A pair of investigators independently carried out the data extraction. Disagreements were resolved by discussion, with input from other investigators. Data extracted from each study included: the first author’s last name, year of publication, study location, study design, characteristics of study population (length of follow-up, sample size), number of ASD participants with or without HDP, effect sizes of the associations, covariates adjusted for in the analysis; diagnostic criteria of HDP and scoring of Newcastle-Ottawa Quality Assessment Scale (NOS).

### Quality evaluation

Quality evaluation of each included study was performed utilizing the Newcastle–Ottawa Scale (NOS) [40]. The assessment consisted of three major categories: selection (four items, one star for each), comparability (one item, up to two stars) and exposure (three items, one star for each). A maximum of nine stars can be given to one study. A final score of seven stars or more was regarded as high quality.

### Statistical analysis

Stata version 12 (Stata Corporation, USA) was used for statistical analyses. Pooled odds ratios (OR) with 95% confidence intervals (CIs) between HDP and ASD were used to estimate effect sizes. The heterogeneity was assessed using both chi-squared test and I^2^ method [41]. When *P* value for chi-squared test was less than 0.10 or I^2^ was more than 50%, there was obvious heterogeneity among those included studies, and random-effect model was used to pool data. If there was no obvious heterogeneity among those included studies, a fixed-effect model was used to pool data. Subgroup analyses were stratified by clinical classification of HDP, maternal age, perinatal complications (preterm birth and premature rupture of membranes), maternal education, geographic area and gender of participants. Sensitivity analysis was performed by omitting one study by turns to test the changes of pooled ORs. Potential risk of publication bias was estimated by inspection of funnel plot. Publication bias was also assessed by Egger’s test [42].

## RESULTS

### Literature search and study characteristics

Detailed search procedures are summarized in Figure 1. A total of 242 individual abstracts for potential studies were identified through literature search. 184 abstracts were excluded because they were irrelevant to the current meta-analysis. Of the 58 remaining clinical studies, 26 were further excluded for lacking of autism patients and 7 were letters, comments, editorials, or reviews, leaving 25 appropriate articles for full-text screening. We scrutinized the 25 articles and excluded 4 articles, including 2 exploring the association between HDP and schizophrenia and 2 showing outcomes as pregnancy complications. Thus, 21 studies with 6,527,652 unique participants met criteria for inclusion in the present meta-analysis. Among these 21 studies, 9 studies assessed the association between gestational hypertension and ASD, 11 assessed the relationship between pre-eclampsia and ASD, and the other 4 assessed the relationship between CHP and ASD. The detailed characteristics of these included studies are shown in Table 1.

**Table 1:** Characteristics of all identified studies

Reference	year	location	Design	Duration	sample size	HDP(+)/ASD(+)	HDP(+)/ASD(-)	OR (95%CI)	adjustment for covariates	Diagnostic criteria	NOS
**Glasson EJ**	2004	Australia	Retrospective cohort	1980–1995	1778	33/465	94/1313	0.99 (0.66–1.50)	Gender, birth year, maternal age, PROM	ICD-9 DSM	7
**Buchmayer S**	2009	Sweden	Case-control	1987–2002	7296	**GH**: 21/1216 **PE**: 39/1216	**GH**: 90/6080 **PE**: 140/6080	GH: 1.04 (0.59–1.81) PE: 1.64 (1.08–2.49)	Maternal age, preterm birth, gender, birth year, delivery hospital	ICD-9 ICD-10	8
**Matsuishi T**	1999	Japan	Prospective cohort	1983–1987	223	2/18	27/205	NR	Maternal age, PROM	DSM-III-R	8
**Stein D**	2006	Israel	Case-control	1970–1998	358	**GH**: 23/206 **PE:** 11/206	**GH**: 17/152 **PE**: 18/152	NR	Gender, maternal education, maternal age, preterm birth, PROM	ICD-VIII DSM-III DSM-IV	5
**Larsson HJ**	2005	Denmark	Case-control	1972–1999	9464	11/364	181/9100	1.54 (0.83–2.86)	Child age, gender, birth year, preterm birth, maternal education	ICD-8 ICD-10	7
**Burstyn I**	2010	Canada	Cohort	1998–2004	216342	27/1122	2747/215220	1.49 (1.00–2.23)	Maternal age, gender	ICD-9	7
**Mann JR**	2010	America	Retrospective Cohort	1996–2002	87677	52/472	5479/87205	NR	Gender, maternal age and education, preterm birth	ICD-9	8
**Walker CK**	2015	America	Case-control	2003–2011	867	**CHP**: 17/510 **Mild PE**: 23/408 **Severe PE**: 26/408	**CHP**: 4/347 **Mild PE**: 9/277 **Severe PE**: 7/277	NR	Child age, gender, geographic area, maternal education	ADI-R ADOS	5
**Gillberg C**	1983	Sweden	Case-control	1962–1980	50	12/25	6/25	NR	Gender; obestetric department	NR	8
**Dodds L**	2011	Canada	Retrospective cohort	1990–2002	129733	106/924	11730/128809	1.24(1.02–1.52)	Gender, maternal age, preterm birth	ICD-9 ICD-10	7
**Mason-Brothers A**	1990	America	Retrospective Cohort	1965–1984	285	17/225	11/60	NR	Gender, PROM, maternal age	DSM-III	5
**Krakowiak P**	2012	America	Case-control	2003–2010	832	**CHP**: 19/517 **GH**: 148/517	**CHP**: 4/315 **GH**: 61/315	NR	Gender, maternal age and education, ethnicity, preterm birth	ADI-R ADOS	7
**Moore GS**	2012	America	Retrospective cohort	1991–2001	5979605	**CHP**: 201/21717 **PE**: 1249/21717	**CHP**: 38158/5957888 **PE**: 257401/5957888;	**CHP**: 1.45 (1.26–1.67) **PE**: 1.42 (1.29–1.57);	Gender, maternal age, ethnicity, birth order	ICD-9	7
**Korzeniewski SJ**	2013	America	Case-control	1984–1987	177	5/12	27/165	NR	Gender, birth year, birthweight, maternal age and education, preterm birth	NR	6
**Hadjkacem I**	2016	Tunisia	Case-control	2014–2014	101	5/50	3/51	NR	Gender, maternal age, gender, preterm birth	DSM-V	6
**Bilder D**	2009	America	Case-control	1998–2002	13320	**CHP**: 1/120 **GH**: 7/120	**CHP**: 63/13200 **GH**: 524/13200	**CHP**:1.752 (0.241–12.738) **GH**:1.466 (0.695–3.231)	Gender, birth year	DSM-IV-TR	7
**Lyall K**	2012	America	Prospective cohort	1989–2003	66445	111/793	6700/65652	NR	Ethnicity, marital status,maternal age	NR	5
**Langridge AT**	2013	Australia	Retrospective cohort	1984–1999	5303	-/727	-/4576	1.25 (0.97, 1.61)	Gender	DSM-IIIR DSM-IV	6
**Polo-Kantola P**	2014	Finland	Case-control	1990–2005	5168	56/1036	147/4132	1.49 (1.10–2.10)	Gender, birth year, birth place, maternal age	ICD-9 ICD-10	7
**Hultman CM**	2002	Sweden	Case-control	1987–1994	2448	24/408	76/2040	1.6 (0.90–2.90)	Gender, birth year, and delivery hospital, maternal age, preterm birth	ICD-9	7
**Say GN**	2015	Turkey	Case-control	2015–2015	180	14/100	6/80	NR	Age, gender	DSM-IV	5

OR, odds ratio; NR, not referred; ICD, International Classification of Diseases; DSM, Diagnostic and Statistical Manual; ADI-R, Autism Diagnostic Interview-Revised; ADOS, Autism Diagnostic Observation Schedule; NOS, Newcastle-Ottawa Quality Assessment Scale; GH: Gestational hypertension; PE: Pre-eclampsia; CHP: Chronic hypertension complicating pregnancy; PROM: Premature rupture of membranes

### Quality assessment

All 21 eligible studies were assessed for quality according to the NOS. The quality of them varied from 5 to 8, with a mean of 6.57 (Table 2). All studies were included in the subsequent analysis.

**Table 2:** Appraisal of methodological quality (Newcastle-Ottawa Scale) of the including studies

Study	Case-cohort representative	Selection of non-exposed control	Ascertainment of exposure	Outcome negative at start	Comparability by design	Comparability by analysis	Outcome assessment	Duration of follow-up	Score
**Glasson EJ**	^*^	^*^	^*^	^*^	^*^	^*^	^*^	×	7
**Buchmayer S**	^*^	^*^	^*^	^*^	^*^	^*^	^*^	^*^	8
**Matsuishi T**	^*^	^*^	^*^	^*^	^*^	^*^	^*^	^*^	8
**Stein D**	^*^	^*^	^*^	^*^	×	^*^	×	×	5
**Larsson HJ**	^*^	^*^	^*^	×	^*^	^*^	^*^	^*^	7
**Burstyn I**	^*^	^*^	×	^*^	^*^	^*^	^*^	^*^	7
**Mann JR**	^*^	^*^	^*^	^*^	^*^	^*^	^*^	^*^	8
**Walker CK**	^*^	^*^	^*^	×	^*^	^*^	×	×	5
**Gillberg C**	^*^	^*^	^*^	^*^	^*^	^*^	^*^	^*^	8
**Dodds L**	^*^	^*^	^*^	^*^	×	^*^	^*^	^*^	7
**Mason-Brothers A**	^*^	×	^*^	×	^*^	^*^	×	^*^	5
**Krakowiak P**	^*^	^*^	×	^*^	^*^	^*^	^*^	^*^	7
**Moore GS**	^*^	×	^*^	^*^	^*^	^*^	^*^	^*^	7
**Korzeniewski SJ**	^*^	×	^*^	^*^	^*^	^*^	^*^	×	6
**Hadjkacem I**	^*^	×	×	^*^	^*^	^*^	^*^	^*^	6
**Bilder D**	^*^	^*^	^*^	×	^*^	^*^	^*^	^*^	7
**Lyall K**	^*^	^*^	×	^*^	^*^	×	^*^	×	5
**Langridge AT**	^*^	^*^	^*^	^*^	^*^	×	^*^	×	6
**Polo-Kantola P**	^*^	^*^	×	^*^	^*^	^*^	^*^	^*^	7
**Hultman CM**	^*^	^*^	×	^*^	^*^	^*^	^*^	^*^	7
**Say GN**	^*^	^*^	^*^	×	^*^	×	^*^	×	5

^*^ Indicates that a feature is present; ×, that a feature is absent. But for comparability by design this checklist awards a maximum of two stars (^**^), one (^*^) or none if the feature is completely absence(×).

### Association of HDP with ASD in offspring

**Figure 2:**
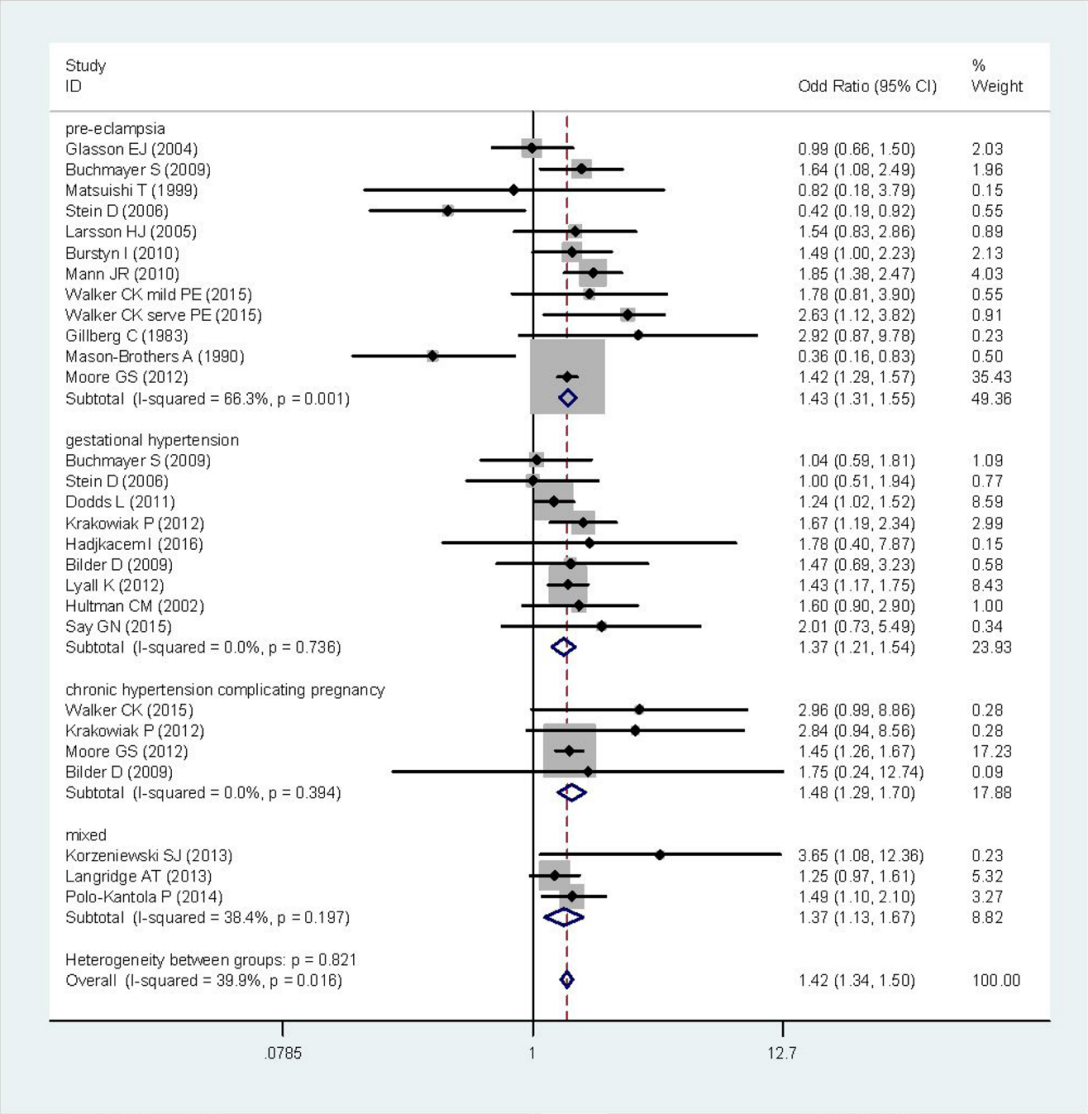
Forest plot of the correlation between HDP and ASD The mixed group include pre-eclampsia, gestational hypertension and chronic hypertension complicating pregnancy.

After summarizing estimates from all available studies, there was a significant association between HDP and incidence of ASD (OR = 1.42, 95% CI: 1.34–1.50) (Figure 2), without obvious heterogeneity (*I*^2^ = 39.9%, *P* = 0.016).

### Subgroup analysis

Significant associations were detected in almost all strata of subgroup analyses according to clinical classification of HDP, maternal age, perinatal complications (preterm birth and premature rupture of membranes), maternal education, geographic area and gender of participants. Results of subgroup meta-analyses are summarized in Table 3.

**Table 3:** Subgroup analysis of the association between HDP and ASD

	Studies	OR	95% CI
**Clinical classification**			
Gestational hypertension	9	1.37	1.21–1.54
Pre-eclampsia	11	1.43	1.31–1.55
Chronic hypertension complicating pregnancy	4	1.48	1.29–1.70
Mixed	3	1.37	1.13–1.67
**Maternal age**			
Higher in the case group	7	1.44	1.33–1.55
Match between case and control	7	1.29	1.11–1.50
Lower in the control group	2	1.42	1.16–1.73
Not mentioned	4	1.50	1.22–1.84
**Preterm birth**			
Higher rate in the case group	7	1.41	1.22–1.63
Match between case and control	3	1.78	1.33–2.37
Higher rate in the control group	1	1	0.51–1.95
Not mentioned	10	1.41	1.32–1.51
**Premature rupture of membranes**			
Higher rate in the case group	1	0.82	0.18–3.76
Match between case and control	3	0.92	0.66–1.28
Higher rate in the control group	1	1	0.51–1.95
Not mentioned	16	1.45	1.36–1.54
**Maternal education**			
Higher in the case group	1	3.65	1.08–12.35
Match between case and control	3	1.90	1.57–2.31
Higher in the control group	2	1.26	0.80–1.99
Not mentioned	15	1.38	1.30–1.47
**Geographic area**			
Asia	3	1.18	0.70–1.99
America	10	1.45	1.36–1.54
Europe	6	1.46	1.16–1.84
Africa	1	1.78	0.40–7.90
Oceania	2	1.17	0.94–1.45
**Gender of participants**			
Male/female higher in case group	8	1.38	1.29–1.48
Match	9	1.68	1.42–1.99
Male/female higher in control group	0	-	-
Not mentioned	4	1.44	1.19–1.74

The mixed group include pre-eclampsia, gestational hypertension and chronic hypertension complicating pregnancy.

### Analysis of sensitivity and publication bias

**Figure 3:**
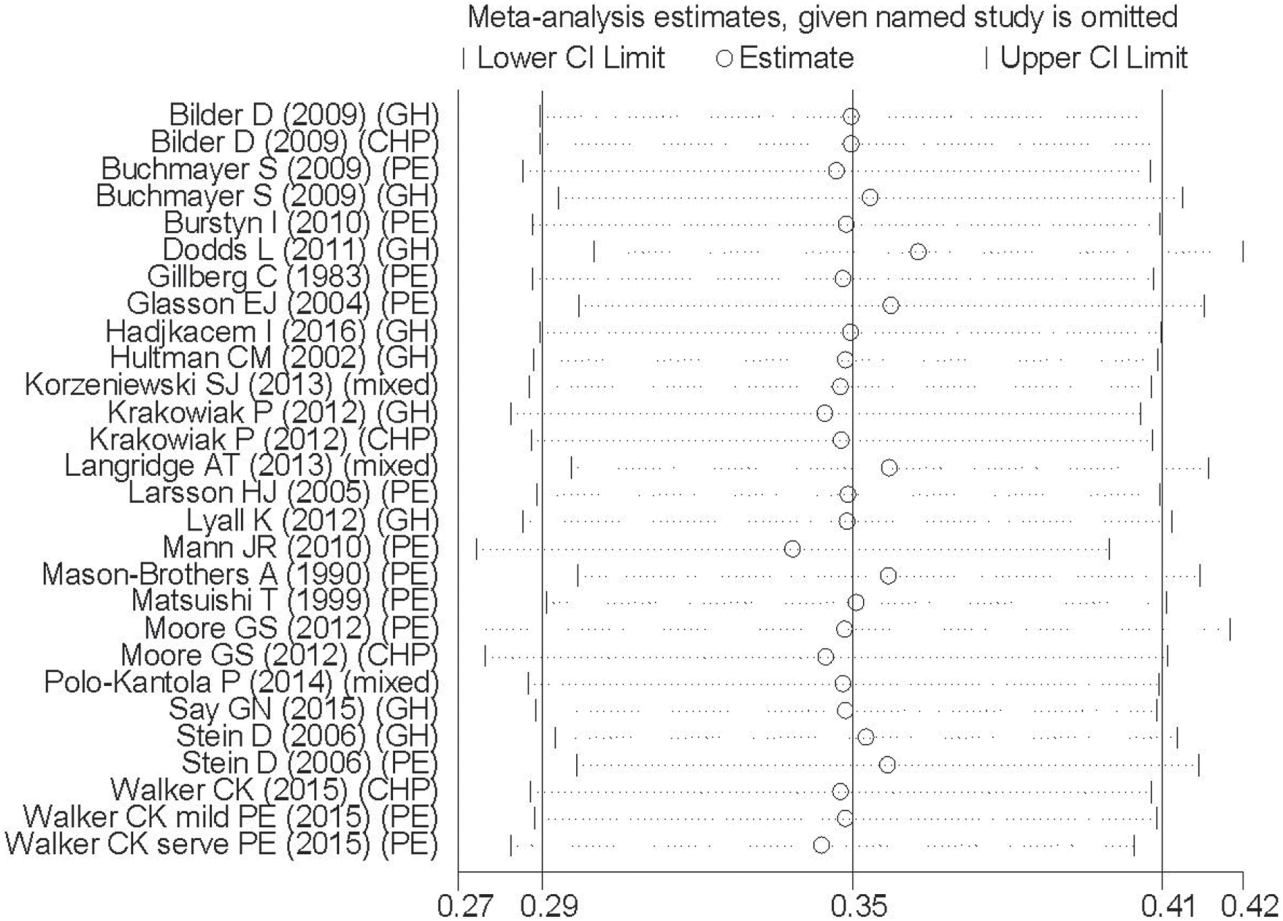
Sensitivity analysis of 21 studies included in this meta-analysis GH: Gestational hypertension; PE: Pre-eclampsia; CHP: Chronic hypertension complicating pregnancy.

**Figure 4:**
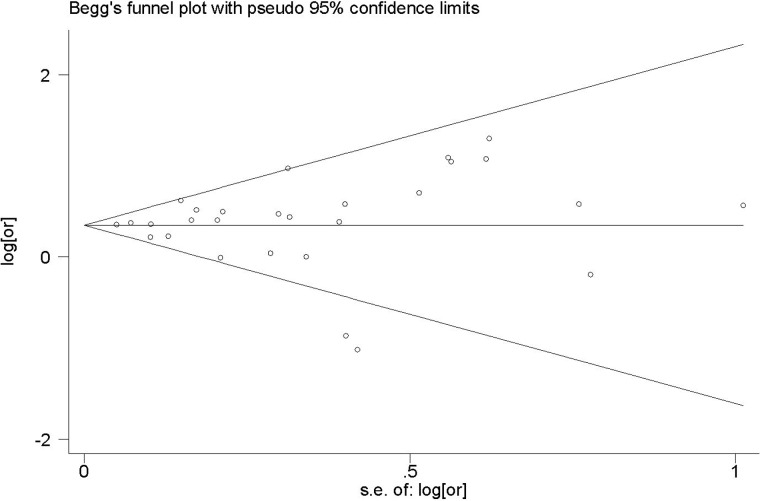
Funnel plot of 21 studies included in this meta-analysis

Sensitivity analysis performed by omitting one study by turns showed all the pooled ORs were statistically significant (Figure 3). In addition, the funnel plot of this meta-analysis seemed to be symmetrical (Figure 4). And the Begg’s test (*p* = 0.441) and Egger’s test (*p* = 0.806) all suggested that there was not a possibility of publication biases that would influence the stability of the results.

## DISCUSSION

Currently, accumulating studies were performed to evaluate the relationship between pregnant complications and child neurodevelopmental disorders based on populations. A previous meta-analysis [43] conducted by Gardener in 2009, suggested that there is insufficient evidence to implicate any one prenatal factor in autism etiology, and non-significant association (OR = 1.01, 95% CI: 0.80–1.27) has been observed between pre-eclampsia/hypertension/edema and ASD. However, fourteen properly designed clinical trials were published from 2009 to 2017. Of these 14 studies, ten indicated a higher incidence of offspring ASD among HDP, while only 4 reported non-significance. Our meta-analysis update the previous review by covering the additional 9 years of publications, indicating that intrauterine exposure to HDP is associated with a 42% increase in the odds of ASD in the pooled estimate.

There is increasing evidence that maternal immune activation (MIA) is associated with a wide array of neurologic and psychiatric disorders [44, 45]. It is well documented that HDP, which is a state of MIA in itself, influences the fetal CNS development through inflammation. Subgroup meta-analyses defined by clinical classification of HDP revealed a slightly higher risk of pre-eclampsia (OR = 1.43) or CHP group (OR = 1.48) than gestational hypertension (OR = 1.37), suggesting the incidence of ASD might be correlated with disease severity. Our results showed that the maternal age may not be a confound factor in the main analysis. The rate of preterm birth was higher in case group, indicating it was a potential confounder in the analysis of HDP and ASD, as most HDP pregnancy must be terminated through cesarean section before full-term. The subgroup analysis of premature rupture of membranes and maternal education didn’t provide powerful evidence due to the limited statistical information. On the other hand, we found that males increased the risk of ASD than females (with an increase in odds by as much as 38%), highlighting that sex-specific considerations will be crucial in exploring the underlying mechanism.

A meta-analysis [46] performed by Wang et al. in 2017 identified about 40 prenatal, perinatal, and postnatal factors which might increase the risk for ASD. However, these factors were examined individually. Therefore, it was still unclear that whether these factors are causal or play a secondary role in the development of autism. Moreover, although pre-eclampsia and gestational hypertension were identified as risk factors for autism in their study, these results were based on 3 or 5 studies, which had potential impact on the overall effect estimates. While in the present study, 9 and 11 studies were selected respectively to explore the association between gestational hypertension/pre-eclampsia and ASD, to draw a more reliable conclusion.

The strength of our meta-analysis, firstly, lies in its large sample size and comprehensive subgroup analysis, which allows a detailed examination of this association by stratifying the data according to clinical classification of HDP, maternal age, perinatal complications , maternal education, geographic area and gender of participants. Secondly, this study provides clinical evidence for the association between HDP and ASD in offspring, indicating that HDP may confer vulnerability to the fetal neurodevelopment. More clinical investigations are necessary to deeply investigate the mechanism, in the hope of illustrating the etiology of ASD and generating new therapies. Thirdly, the results from our sensitivity analyses suggest that our main results were credible to inclusion of studies and were not driven by a single study. There was insufficient statistical evidence of publication bias based on Egger’s test. However, this meta-analysis also has some inherent limitations warranting discussion. First, blood pressure grade of HDP and drug treatment have been seldom mentioned in the included studies. Hence, the between-study heterogeneity of blood pressure or treatment strategy could be potential confounders precluding rigorous evaluation. Second, the pooled estimates were originated from 12 case-control, 7 retrospective cohort and only 2 prospective studies. The NOS scores of included studies range from 5 to 8, with a mean of 6.57, indicating moderate quality according to NOS scale (moderate quality scored 4–6). There is a compelling need for larger-size, multi-center and higher-quality prospective studies with unified criteria to obtain more persuasive conclusions. Finally, our meta-analysis is not suitable for worldwide generalizability due to the geographic restriction of the selected studies, which were mostly comprised of participants from America or Europe. Future clinical investigations performed in other regions are highly needed.

In summary, the present comprehensive meta-analysis suggests that intrauterine exposure to hypertensive disorders during pregnancy might be a risk factor for ASD in offspring. Identifying the infants most at risk for ASD is crucial to allow for preventative interventions from birth [47]. One intriguing possibility is making use of the placenta, a readily accessible tissue at birth but normally discarded, to serve as a non-invasive method to predict abnormal fetal brain development. It would be worthwhile to explore the association between placental pathologies and ASD of offspring in the future, which may lead to targeted surveillance or prevention approaches, in order to decrease the risk and incidence of ASD.
